# What presents to a rural district emergency department: A case mix

**DOI:** 10.4102/phcfm.v12i1.2275

**Published:** 2020-07-28

**Authors:** Nadishani T. Meyer, Gareth D. Meyer, Charles B. Gaunt

**Affiliations:** 1 Jabulani Rural Health Foundation, Mqanduli, South Africa; 2 Zithulele Hospital, Mqanduli, South Africa

**Keywords:** case mix, rural hospital emergency department, rural health, rural medicine, Specialist Emergency Medicine

## Abstract

**Background:**

There is little information available on the range of conditions presenting to generalist run rural district hospital emergency departments (EDs) which are the first point of acute care for many South Africans.

**Aim:**

This study aims to assess the range of acute presentations as well as the types of procedures required by patients in a rural district hospital context.

**Setting:**

Zithulele is a 147-bed district hospital in rural Eastern Cape.

**Methods:**

This is a cross-sectional study assessing all patients presenting to the Zithulele hospital emergency department from 01 October 2015 to 31 December 2015. Data collected included the triage acuity using the South African Triage Scale system, patient demographics, diagnosis, outcome and procedures performed. Diagnoses were coded retrospectively according to the international statistical classification of diseases and related health problems version 10 (ICD 10).

**Results:**

Of the 4 002 patients presenting to the ED during the study period, 2% were triaged as emergencies and 45% as non-urgent. The most common diagnostic categories were injuries, infections and respiratory illnesses respectively. Diagnoses from all broad categories of the ICD-10 were represented. 67% of patients required no procedure. Diagnostic procedures (*n* = 877) were more prevalent than therapeutic procedures (*n* = 377). Only 2.4% of patients were transferred to a referral centre acutely.

**Conclusion:**

Patients with conditions from all categories of the ICD-10 present for management at rural district hospitals. Healthcare professionals working in this setting need to independently diagnose and manage a wide range of ED presentations and execute an assortment of procedures.

## Introduction

### Background and rationale

The general term ‘case mix’ refers to the systematic grouping of types of presenting conditions and the types of procedures performed in response to these presentations to health centres.^1^ Alphanumeric codes are the preferred method of classification of the wide range of presentations in order to allow for comparability between institutions. Case-mix analyses inform the application of policy and assist regional and national health managers with programme planning and staff and resource allocations in specific health areas.^2^ They also assist managers at institutional level to allocate resources appropriately, and identify clinical training needs among staff.

In South Africa, there is no easily accessible central database for presentations to Emergency Departments (EDs). Government facilities report data on the District Health Information System (DHIS), which uses very broad categories of presenting conditions. As a result, small observational studies represent a credible method of gaining a more complete understanding of what presents to South African hospital EDs. Similar problems have been encountered in developed countries such as Canada and Australia, which have grappled with the complicated process of characterising their rural hospital EDs by performing small observational studies such as this one.^4^

There are very few research studies examining the case mix of EDs in South Africa. Most studies conducted have been in the Western Cape, assessing the case mix in Community Health Centres in the Cape Town metropole,^5^ an urban district hospital,^6^ and an urban provincial hospital.^7^ There were two other studies conducted in urban district EDs in Middelburg^8^ and Bloemfontein.^9^ We could not find any studies published on the case mix in respect of rural district hospital EDs in South Africa. This is a significant gap in the literature as the rural ED has specific contextual characteristics, particularly with regard to patient access, transfer and lack of resources, which may lead to presumptions with regard to how these factors affect the case mix in rural EDs.

District level emergency care consists of three tiers including primary healthcare clinics, community health centres and district hospitals. Primary healthcare clinics are nurse led and operate only during working hours on weekdays. Community health centres have doctors available during working hours and offer nurse-led emergency care after hours. Complicated patients are referred to a district hospital ED. In rural areas, the district hospital ED is the only facility with a 24-h doctor-led emergency service. It is characteristically run by general practitioners who are expected to diagnose and manage any presenting conditions independently. Specialist care may be accessed by transferring a stabilised patient to a higher referral health centre (secondary or tertiary level facility) if more complex interventions are required. The rural district hospital ED thus functions as an important bridge between the primary healthcare system and the specialist secondary and/or tertiary level of emergency care. According to the World Bank, approximately 35% of South Africans lived in rural areas in 2016,^10^ which emphasises the degree of importance of the rural district hospital EDs when assessing emergency care provision in South Africa.

This study aims to highlight the range of conditions presented for evaluation and management at a rural district hospital. We hope it will inform decisions regarding resource allocation and skills development, as well as educate prospective rural health workers with regard to the diagnostic and practical skills which would be required to thrive in this setting.

### Objectives

1. To assess the case mix, including diagnoses, acuities and other characteristics of patients presented to a rural district hospital ED.
2. To characterise the ED to inform resource allocation and skills development at Zithulele Hospital.

## Methods

### Study design

This cross-sectional study included all patients presenting to the emergency department of Zithulele district hospital for the period 01 October to 31 December 2015.

### Setting

Zithulele Hospital is a 147-bed rural district hospital situated in a deeply rural part of the Eastern Cape province and serves a population of approximately 130 000 people over a 1042 km^2^ area. It serves as a referral centre for 16 primary healthcare clinics and two community health centres. The hospital is general practitioner led with specialist care accessible at the tertiary level referral centre, which is roughly 90 km away. Few patients have access to private vehicles and most walk to the main road and travel by mini-bus taxi to the hospital. The mini-bus taxis operate only during daylight hours. Transportation at night necessitates the hire of a private vehicle which is a source of catastrophically high costs to a patient or family.^3^ Two ambulances service the area and transport patients between facilities or from the scene of motor vehicle accidents and rarely have the capacity to collect patients from their homes.

### Study population, sample size and sampling

All patients presenting to the Zithulele hospital ED during the three-month study period comprised the study population. No further sampling was performed.

### Data collection

The South African Triage Scale was used in the ED. Institution-specific triage forms were already in use at the time of the study. The triage nurse documented the arrival time, triage time, referral clinic (or whether self-referred), the patients’ physiological status, final triage colour, presenting complaints, and demographics. The attending doctor then recorded the time seen (by doctor), final diagnosis, procedures performed and outcome of consultation, i.e. admitted to ward, discharged, transferred, deceased or absconded. Information was collected from triage forms and tabulated in a Microsoft Excel® spreadsheet and stored on a password-protected laptop.

### Data analysis

Diagnoses were coded by the authors retrospectively using the international statistical classification of diseases and related health problems version 10 (International Classification of Diseases ICD 10) according to the diagnosis recorded on the triage form by the attending doctor. Descriptive data was analysed using Microsoft Excel.

### Ethical consideration

Ethics approval was obtained prior to data collection from the University of Cape Town Human Research Ethics Committee (575/2015). Verbal consent for the use of data from the triage forms was obtained from every patient at the time of triage (or from the next of kin in the case of underage or unconscious patients) by the triage nursing professional.

## Results

A total of 4011 patients presented to the Zithulele ED during the study period. Eight of the patients were seen by medical officers without being triaged and one patient was dead on arrival. These patients were excluded from the study, leaving 4002 patients for analysis.

Of the 4002 patients, 3681 had a recorded gender, of which 1548 (38.7%) were male and 2133 (53.3%) were female. Three hundred and forty-nine (16%) of the female patients were recorded as pregnant. Females predominated over males in every age category other than children aged three to 12 years.

**FIGURE 1:**
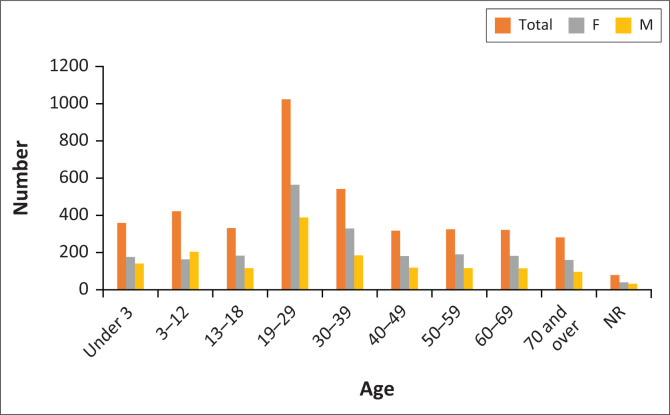
Age and gender distribution of presenting patients.

A total of 3923 patients had recorded ages: 3142 (80%) patients over 12 years of age, 359 (9%) patients aged three to 12, and 422 (11%) patients under three years. Patients aged 19 to 39 years made up the largest group of adults at 56% (*n* = 1566), while patients over 60 years of age constituted the next largest group at 21% (*n* = 603). Figure 1 shows the distribution of presenting patients by age and gender.

The range of acuities of the presenting patients as determined by the South African Triage Scale were 66 (1.6%) Red/Emergency patients; 605 (15%) Orange/Very urgent patients; 1500 (37.5%) Yellow/Urgent patients; and 1831 (45.8%) Green/Non-urgent patients.

The HIV status of 2908 patients (72.7% of the cohort) was recorded. Five hundred and twenty (17.9%) patients were recorded as HIV positive and 1437 (49.4%) were recorded as HIV negative, while an additional 951 (32.7%) were recorded as unknown.

Of the patients where the referral source was recorded (*n* = 3 474), 32.2% were self-referred patients, with the remainder referred by local primary care structures. Five hundred and seven self-referred low acuity (SATS green) patients presented during the study period.

**FIGURE 2:**
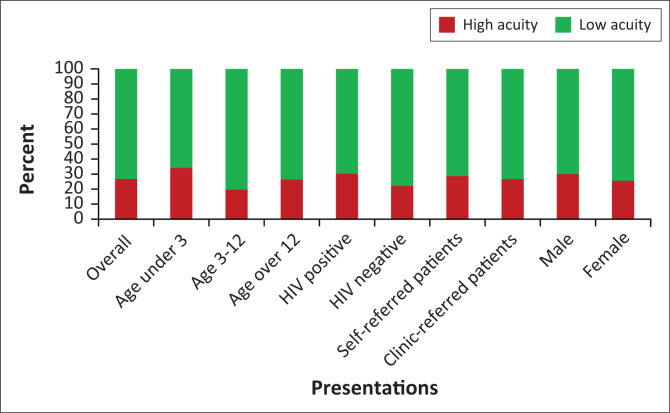
Acuity presentations by age, gender, human immunodeficiency virus (HIV) status, clinic/self-referral status.

As shown in Figure 2, there were statistically significant increases in higher acuity presentations (red and orange patients) in HIV-positive patients when compared with HIV-negative patients (RR 1.36, 95% CI 1.12–1.67, *p* = 0.002); patients under three years when compared with those over 12 years (RR 1.29, 95% CI 1.06–1.57, *p* = 0.012); and male patients when compared with female patients (RR 1.17, 95%, CI 1.027–1.34, *p* = 0.02). The age category three to 12 was significantly less likely to be triaged as high acuity compared to the over-12 age category (RR 0.75, 95% CI 0.57–0.98, *p* = 0.03).

There was no statistically significant difference in high acuity presentations between patients who were self-referred when compared with those referred by local clinics (*p* = 0.34).

During the study period 3 957 outcomes were recorded upon disposition from the ED: 798 (20%) patients were admitted; 2901 (73%) patients were discharged; 8 (0.2%) patients demised in the ED (discussed further in Table 1); 94 (2.3%) patients were transferred to a higher level of care; and 156 (4%) absconded. Of the patients who were transferred, the most common diagnoses were head injury (*n* = 8), wrist and lower limb fractures (*n* = 8), appendicitis and acute abdomen (*n* = 7) and stroke (*n* = 4).

**TABLE 1:** Top 10 diagnoses by age categories and overall for emergency department presentations.

Rank	Overall	Under 3	3–12	> 12	19–29	> 60
1	Unspecified injury 223	AGE	Unspecified injury	Unspecified injury	Unspecified injury	LRTI
2	LRTI 166	LRTI	Wrist and hand and wrist	PTB	Normal Pregnancy	COPD
3	AGE 152	PTB	PTB	LRTI	Assault	CCF
4	PTB 143	Poisoning	Supracondylar fracture	Normal pregnancy	PTB	AGE
5	Normal pregnancy 88	URTI	LRTI	AGE	UTI	PTB
6	UTI 87	Febrile convulsions	AGE	UTI	Epilepsy	Stroke
7	Epilepsy 64	Scabies	Bilharzia	Assault	Spontaneous Abortion	UTI
8	Assault 63	Abscess	Convulsions	Epilepsy	AGE	Unspecified injury
9	Abscess 55	Dysentery	Epilepsy	COPD	Pre-Eclampsia	Hypertension
10	COPD 54	Unspecified injury	Asthma	CCF	Lower limb Fracture	Diabetes

AGE, Acute Gastroenteritis; CCF, Congestive Cardiac Failure; COPD, Chronic Obstructive Pulmonary Disease; LRTI, Lower Respiratory Tract Infection; PTB, Pulmonary Tuberculosis; URTI, Upper Respiratory Tract Infection; UTI, Urinary Tract Infection.

Eight patients demised in the ED; data was missing for three of the eight patients. From analysis of the available data, however, no clear pattern of triage acuity, age, gender, or diagnosis emerges when assessing these cases. One of the cases was a one-year old female who died of acute gastroenteritis. The other four cases were all adults whose causes of death included severe sepsis (aged 20, triaged orange); smoke inhalation (aged 50, triaged red); cardiac dysrhythmia (aged 17, triaged yellow); and meningitis (aged 33, triaged orange). All of the cases presented after hours.

**FIGURE 3:**
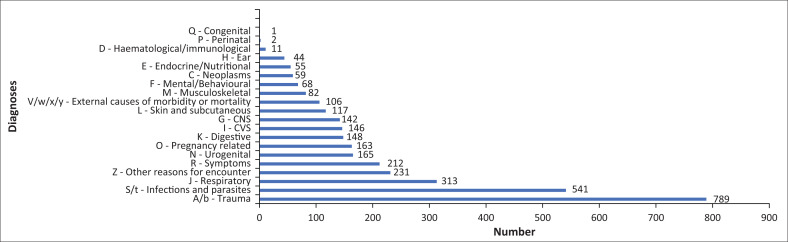
Diagnoses by International Classification of Diseases ICD-10 category of patients presenting to the emergency department.

A total of 562 patients had no diagnosis recorded and a further 81 had illegible diagnoses. Four hundred and twenty-one different diagnoses made in the ED were identified as per ICD-10 coding. The most common single diagnosis was ‘unspecified injury’, followed by ‘lower respiratory tract infections’. The 10 most common diagnoses by age are presented in Table 1. All broad categories of disease classification in the ICD-10 were represented as shown in Figure 3.

**FIGURE 4:**
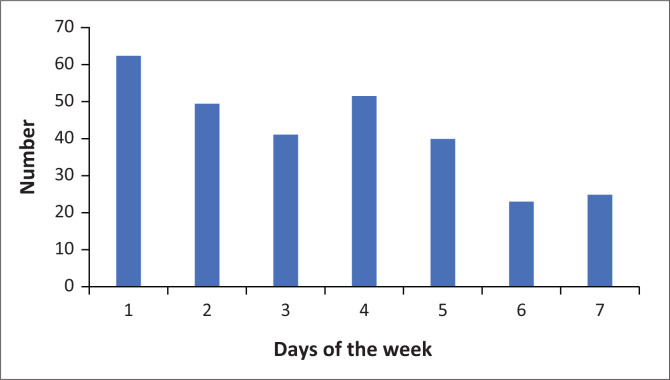
Average number of patients presenting by day of the week.

**FIGURE 5:**
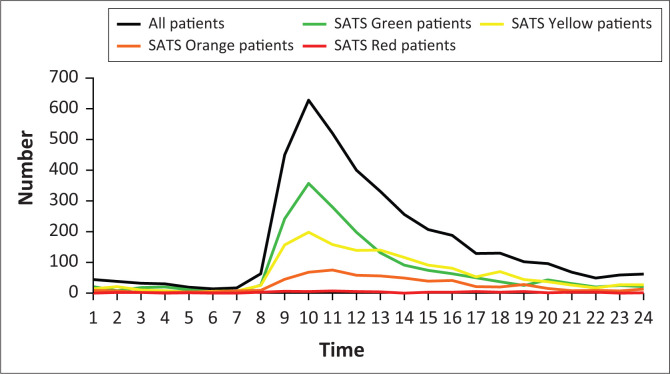
Arrival time of patients presenting to the emergency department by South African Triage Score (SATS) acuity.

During the study period, there were clear differences in patient load depending on the day of the week, with significantly lower average numbers per day on the weekend as compared to weekdays; and the highest number of patients seen on Mondays (Figure 4). The average overall peak arrival time was between 09:00 and 10:00, the bulk of whom were low-acuity patients. The vast majority of patients presented between 07:00 and 17:00. Patients with higher acuity presented less predictably with no clear peak noted in emergency (SATS red) patients (Figure 5).

Data collection for procedures performed was reliant on doctors recording the procedures required at the point of consultation. Concerns regarding the completeness of the records have previously been noted as a limitation. Sixty-nine point seven per cent of patients (*n* = 2791) were recorded as having had no procedure performed. One hundred and forty-two triage forms lacked a note regarding whether any procedure had been performed. X-rays were requested for 14% (*n* = 556) of presentations.

Of the remaining 1069 forms with documented procedures, 1250 procedures were recorded. Diagnostic procedures constituted the majority of procedures performed, including phlebotomy (*n* = 507) and ultrasound (*n* = 246). Of the 4002 patients, 22.5% (*n* = 904) required only one procedure, 3.8% (*n* = 152) required two procedures and only 0.3% (*n* = 13) required three or more procedures.

## Discussion

Understanding emergency care in South Africa requires an appreciation of the specific case mix of the rural district ED. In the absence of a standardised national database, profiling a single ED over a specified period of time allows for a better understanding of the resources, both human and physical, required for optimal service in a given context.

**TABLE 2:** Procedures performed in the Emergency Department during the study period.

All documented procedures	*N*
**Diagnostic Procedures**	**873**
Phlebotomy	507
Ultrasound	246
Lumbar puncture	50
Fine Needle Aspirate Biopsy	23
Electrocardiogram	23
Tissue Biopsy	12
Pleural tap	7
Ascitic tap	5
**Therapeutic Procedures**	**377**
Wound suture	155
Plaster of Paris	75
Intravenous cannulation	42
Incision and drainage of abscess	24
Sedation	23
Manipulation under anaesthesia	22
Intercostal drain insertion	14
Foreign body removal	11
Wound care	7
Intubation	3
Cardioversion	1

A slightly higher percentage of women presented to the ED, which is in keeping with local census data and may be linked to local employment scarcity, resulting in many men being migrant labourers. Age distribution followed patterns noted in other South African studies, with the majority of patients presenting being young adults aged 19 to 39. This may be explained by the burden of trauma, pregnancy-related illness and HIV affecting this age group.

Acuity followed the expected distribution, with only 2% of patients presenting as emergencies (SATS red) and non-urgent cases (SATS green) accounting for 45% of presentations. A wide variety of reported low acuities presentations exists in previous South African ED profiles, with rates of up to 65%.^5^ Access to primary care in the ED catchment area is variable across the country and may account for these differences. Rates of emergency presentations, however, are consistently similar at under 5% in profiles from institutions using the SATS in South Africa.^4,5,6^

The ED encounter is a good opportunity to offer testing for HIV. A recently published article from the Eastern Cape in South Africa suggests that a high percentage of patients, if offered voluntary testing, will agree to be tested.^12^ At least 951 patients were recorded as having unknown HIV status, which we consider to be missed opportunities.

Thirty-two per cent of patients were self-referred, which is lower than some South African profiles which have reported up to 88.2%.^6^ A large geographic drainage area and relatively high transport costs make access to the hospital difficult and bypassing of primary care institutions less attractive. However, the absence of after-hours access to a primary healthcare facility for most people makes some self-referral inevitable.

The vast majority of patients (98%) presenting to the ED were cared for at our institution with an acute transfer rate of only 2%. Reasons for transfer to a specialist referral centre, as elucidated by the diagnoses of the patients who were transferred, was for definitive surgical and/or neurosurgical or orthopaedic surgical management and for more specialised diagnostic imaging, particularly computed tomography (CT) scans. This is a crude indication of the massive emergency care patient load carried at district level and the need for more Specialist Emergency Medicine involvement in guiding and improving care in rural EDs. Rates of patient deaths were very similar to other South African studies at 0.2%. No clear pattern of risk factors for death in the ED was identified.

A broad range of diagnoses was noted in the dataset, with diagnoses from all categories in the ICD-10 coding system. Doctors and nurses working in a rural ED need to be competent at independent management and diagnosis of a wide range of conditions.

Assessment of the most common diagnoses clearly demonstrates the triple burden of trauma, infectious diseases and chronic illness faced by South African patients. Age-specific common diagnoses elucidate some notable features. There is a predominance of infectious diseases in patients under three; injuries in patients three to 12 years old; trauma and pregnancy-related illness in young adults; and chronic illnesses in adults over 60. Trauma is consistently the most common category of disease in ED profiles from other institutions globally. Within our study, 23% of diagnoses were categorised as trauma related, which is marginally lower than in other South African series, reported at between 23% and 48%.^4,5,7^ It is, however, important to note that although trauma constituted the most common category at Zithulele, 78% of presentations are necessarily non-trauma related if a binary classification is used.

Peak patient load times vary quite remarkable among South African EDs. In our setting, extremely high unemployment rates, no public transport available at night and limited access to primary care over weekends most likely contribute to our Monday morning peak time, compared to more urban settings, where employment and transport may contribute to a 16:00 peak, which is absent in our ED.^4^ The reliance on public transport which, given our geography, tends to run towards the hospital in the morning and away in the afternoon, also explains the vast predominance of mid-morning arrivals. The same factors have made it impossible to institute a booked appointment system thus far.

Patient load of on average 1334 patients per month was lower than other described patient loads in South African hospitals (a range of 1528 to 3399 patients per month).^4,5,7^ This lower number of presenting patients may reflect issues with access or the presence of doctor outreaches to local clinics.

Diagnostic procedures predominated, with ultrasound noted as the second most common procedure performed. This is in keeping with the growing use of point of care ultrasound in EDs internationally. The majority of low acuity patients had no procedure performed, which implies that care could have been delivered at primary care level if a doctor had been available.

This type of case-mix evaluation is also useful as a monitoring and evaluation tool to identify areas that may be targeted to improve service delivery. In our setting, it became evident that we had many missed opportunities for HIV testing in the ED; therefore, doctors and nurses working in the ED were encouraged to be more intentional about offering HIV testing to patients whose status was not known. Analysis of the average number of patients presenting per day demonstrated that lower numbers tended to present on a Wednesday, which prompted staff to book small procedures such as removal of lumps and bumps on an elective operative slate on Wednesdays. Lastly, analysis of the average times of presentation of patients to casualty highlighted a clear peak between 09:00 and 13:00, and for this reason, an extra doctor was assigned to cover a shift between 11:00 and 14:00 to ensure that patients continued to be seen timeously.

The main limitation are incomplete triage and/or data forms with missing data, as the investigators relied on nurses and doctors working in an operational clinical setting to fill out all the information required on the triage forms meticulously. A total of 526 of the 4002 forms did not have a documented diagnosis, and it is suspected that many more procedures were performed than were documented on the forms. A second limitation was the decision to use ICD-10 to code the diagnoses retrospectively. It was thought to be a benefit in that it conferred generalisability, as the ICD-10 coding system is widely utilised; however, as our doctors are unfamiliar with the coding system, which is not used routinely in our setting, there were some recorded diagnoses that were challenging to code accurately retrospectively. A third limitation is the ability of this small snapshot of a single rural ED to confer generalisability adequately to the case mixes of other small rural EDs. Additionally, the study period included the Christmas and New Year periods, which anecdotally have higher trauma rates. This may have skewed the data. Ideally, more studies on case mixes from other rural EDs will be conducted to add to the knowledge base.

## Conclusion

There is a large variety of conditions presented to management at rural district hospitals. Healthcare professionals working in these institutions need to be able to diagnose and manage a wide range of ED presentations independently and to execute a wide range of procedures comfortably in order to work in this setting.
